# Sun protection and occupation: Current developments and perspectives for prevention of occupational skin cancer

**DOI:** 10.3389/fpubh.2022.1110158

**Published:** 2022-12-23

**Authors:** Cara Symanzik, Swen Malte John

**Affiliations:** ^1^Institute for Interdisciplinary Dermatological Prevention and Rehabilitation (iDerm), Osnabrück University, Osnabrück, Germany; ^2^Department of Dermatology, Environmental Medicine and Health Theory, Institute for Health Research and Education (IGB), Faculty of Human Sciences, Osnabrück University, Osnabrück, Germany

**Keywords:** exposure, prevention, occupational, skin cancer, solar, sun protection, sunscreen, ultra-violet radiation

## Abstract

A substantial proportion of all reported occupational illnesses are constituted by skin cancer, making this disease a serious public health issue. Solar ultra-violet radiation (UVR) exposure is the most significant external factor in the development of skin cancer, for which the broad occupational category of outdoor workers has already been identified as high-risk group. Sun protection by deploying adequate technical, organizational, and person-related measures has to be understood as a functional aspect of workplace safety. To prevent skin cancers brought on by—typically cumulative—solar UVR exposure, outdoor workers must considerably lower their occupationally acquired solar UVR doses. Estimating cumulative sun exposure in outdoor workers requires consideration of the level of solar UVR exposure, the tasks to be done in the sun, and the employees' solar UVR preventive measures. Recent studies have highlighted the necessity for measures to enhance outdoor workers' sun protection behavior. In the coming decades, occupational dermatology is expected to pay increasing attention to sun protection at work. Also, the field of dermato-oncology will likely be concerned with sky-rocketing incidences of occupational skin cancers. The complete range of available alternatives should be utilized in terms of preventive actions, which seems pivotal to handle the present and future challenges in a purposeful manner. This will almost definitely only be possible if politicians' support is effectively combined with communal and individual preventive actions in order to spur long-term transformation.

## 1. Introduction

The quantity of individuals diagnosed with skin cancer has progressively increased over the past few decades (1, 2). In this day and age, a substantial proportion of all reported occupational illnesses are constituted by skin cancer (3), making this disease a serious issue of public health all over the globe—especially in fair skinned populations. Solar ultra-violet radiation (UVR) exposure is the most significant external factor in the development of skin cancer (4). The broad occupational category of outdoor workers with direct and indirect occupational solar UVR exposure has already been identified as high-risk group for the development of occupational skin cancer (3, 5, 6). In terms of the number of employees exposed and incidence, solar UVR is the most important occupational carcinogenic exposure (7–9), as solar UVR is the leading cause of non-melanoma skin cancer (NMSC), more precisely referred to as keratinocyte carcinoma (KC), which manifests as actinic keratosis (AK, intra-epidermal SCC), invasive cutaneous squamous cell carcinoma (SCC), and/or basal cell carcinoma (BCC) (10–12). Despite the fact that millions of employees worldwide are exposed to the occupational carcinogenic exposure represented by solar UVR for a huge fraction of their working hours, occupational safety and health directives and legislation in many countries around the world still do not acknowledge this work-related risk factor (10, 13, 14). Moreover, no particular occupational exposure threshold values are broadly accepted; however, the International Commission on Non-Ionizing Radiation Protection (ICNIRP) has proposed an occupational solar UVR exposure limit equivalent to 1.0–1.3 Standard Erythema Doses (SED) per day; 1 SED equals 100 J/m^2^ of the biologically weighted erythema action spectrum (14, 15).

Sun protection (e.g., by deploying adequate technical, organizational, and person-related measures) has to be understood as a crucial aspect of occupational safety. Outdoor workers must considerably lower their occupationally acquired solar UVR doses in order to prevent skin cancers brought on by—typically cumulative—solar UVR exposure. Estimating cumulative sun exposure in outdoor workers requires consideration of the level of solar UVR exposure, the tasks to be done in the sun, and the employees' solar UVR preventive measures. Recent studies have revealed a low health literacy in outdoor workers, thus highlighting the necessity for (educational) measures to enhance outdoor workers' sun protection behavior (16). As yet, outdoor workers' particular demands for improved sun protection behavior are frequently disregarded. Risk perception and attitudes regarding sun protection strategies are likely to impact practical sun protection behavior at work (17). In the coming decades, occupational dermatology is expected to pay increasing attention to sun protection at workplaces (3). Also, the field of dermato-oncology will likely be concerned with sky-rocketing incidences of occupational skin cancers, as the global need for not only preventive measures against the constantly rising number of skin cancer cases but also their treatment is high (2). Against this background, this article seeks to present and evaluate current developments and future implications for the prevention of occupational skin cancer.

## 2. Skin cancer by solar ultraviolet radiation (UVR)

### 2.1. Outdoor workers as high-risk group

Outdoor workers are exposed to high amounts of solar UVR due to the fact that they spend most of their work time in the open (18). Thus, they are strikingly more likely to acquire KC than the general population; risk is increased at least 2-fold, with SCC being the cancer most directly related to cumulative UVR exposure (10, 19–21). Examples for occupations with a high share of outdoor work and direct as well as indirect occupational solar UVR exposure are construction workers, agricultural workers, gardeners, child carers, firefighters, police officers, bath attendants, sports people, street vendors, and zoo workers (22).

### 2.2. Malignant melanoma (MM)

Although UVR exposure has been associated with malignant melanoma (MM), intermittent UVR exposure and particularly UVR exposure in children as well as genetic predispositions appear to play a more significant role (23). Even though several recent studies revealed a potential association between chronic occupational sun damage and some MM subtypes [i.e., lentigo maligna melanoma (LMM)] the correlation between (cumulative) occupational solar UVR exposure and MM is seen as not conclusive (11, 24).

### 2.3. Non-melanoma skin cancer (NMSC)

Globally, NMSC is increasing, especially among outdoor workers. Employees in this occupational group frequently experience severe actinic damage, which results in a chronic condition with a great number of newly emerging lesions and calls for ongoing treatment (3). Outdoor workers are commonly exposed to daily solar UVR doses that are regularly five times higher than the ICNIRP exposure guidelines and are exposed to solar UVR doses that are at least 2–3 times more than those of indoor workers (14, 15, 18, 25–30). After years of cumulative solar UVR exposure, epidemiologic studies show a notably high frequency of both BCC and SCC among outdoor workers, suggesting a significant relationship between occupational solar UVR exposure and the prevalence of NMSC (19, 20, 31, 32). Given the long latency period of up to 20-years between exposure and (chronic) illness, the disease frequently remains “hidden” to the patient, just as the causing UVR. Over 80% of cases affect those 60 years of age and older (33), with the exception of outdoor workers who are immunosuppressed and frequently develop AK/SCC very early. The prevalence of occupational NMSC is anticipated to increase over time as a result of the demographic shift, placing an even higher burden on global health care needs and insurance systems (3). At this juncture it should be mentioned that NMSC, however, is one of the few cancers that is easily detectable and recoverable; but most importantly even completely avoidable (3).

The identification of particular at-risk groups among outdoor workers has benefited from the use of objective dosimetric measurements (3, 26, 28–30). The occupation and responsibilities connected with this activity within the industrial sector were found to be more important in determining the amount of solar UVR exposure experienced at work than the industrial sector as a whole (3, 29, 30). As a result, the German statutory social accident insurance (DGUV) adopted the “Wittlich's algorithm,” a mathematical model, which evaluates individual occupational lifetime solar UVR exposure based on the gathered dosimetric data. The “Wittlich's algorithm” is currently used to enhance prevention strategies, healthcare options, and compensation for impacted workers (29). It is based on the idea that if a person's lifetime solar UVR exposure increases by 40% as a result of their job, their risk of developing AK/SCC doubles. Consequently, occupational influences are taken into consideration, and skin cancer that develops in body parts exposed to solar UVR at work is recognized as an occupational disease that is eligible for compensation. Given that the extensive dosimetric measuring campaign in Germany by Wittlich et al. with over a thousand outdoor workers for three full April to October periods revealed surprisingly high occupational exposures of up to 600 SED per summer period (30), outdoor workers typically meet the aforementioned requirements if they work for more than 10 years full-time in a high-risk profession.

## 3. Prevention of (occupational) skin cancer by solar UVR

### 3.1. Sun protection as occupational safety measure

Sun protection is generally a component of workplace safety. In the main, a significant reduction in occupationally acquired UVR doses is necessary for outdoor workers to prevent skin malignancies brought on by solar UVR exposure. The amount of solar UVR exposure, the specific outside activities, and the employees' solar UVR preventive behaviors all play a role in understanding the cumulative sun exposure for outdoor workers. Recommendations incorporate **t**echnical, **o**rganizational, and person-related measures and adhere to the so-called hierarchical TOP concept. In this sense, technical measures are the first to be used and seek to limit exposure to sunlight. This spans all types of shade, including sun sails and weather protection tents. Organizational measures follow technical measures and are used to avoid outside work while the sun is particularly bright. Even changing working hours to avoid the midday sun might be crucial for occupational safety. This is especially true in Europe between the months of April and September from ~11 a.m. to 4 p.m. It is advised to move working hours to the morning, take pauses in the shade, and, if possible, complete individual job tasks generally in the shade. Lastly, person-related measures follow the aforementioned measures and include supporting outdoor workers with appropriate Personal Protective Equipment (PPE)—which in Germany must be funded by the employer. Adequate PPE spans sunglasses with wide, solar UVR filtering lenses, protective clothing with a UVR protection factor (UPF; such as long-sleeved shirts, but “better a T-shirt than no shirt,” and pants), and headgear (such as broad-brimmed helmets or hats with sun shields as well as ear and neck guards) (34, 35).

Sunscreens in itself are not classified as PPE. They should, however, be used in situations where other forms of protection in terms of other measures are impractical (i.e., on the back of the hands and in the facial area). Adequate sunscreens must block UV-A and UV-B rays (i.e., have a so-called broad-spectrum UV filter), have a Sun Protection Factor (SPF) of at least 30, but preferably 50+, and be water- and sweat-resistant as well as simple to apply so that they can be reapplied regularly during the entire day (i.e., ideally every 2 h) in adequate amounts of 2 mg/cm^2^ (34–37). The so-called “two finger rule” can be helpful in this regard; it suggests applying this amount, namely 2 mg/cm^2^, of sunscreen to certain areas, such as the head, neck, and face, by squeezing two strips of sunscreen from the tip to the base of the index and middle fingers.

### 3.2. Primary, secondary, and tertiary prevention

Primary prevention is defined by the International Agency for Research on Cancer (IARC) as any preventive action that reduces the risk of developing cancer in humans (35), with the collective level and the individual level being further sub classified (34). Primary prevention shouldn't be restricted to the corporate sphere; rather, it could be a component of a larger strategy that also includes institutional, governmental, and societal preventative policies and initiatives, as well as the adoption of norms, standards, and prevention-related initiatives (34, 38). The first stage in primary prevention in the workplace is the establishment of an efficient risk assessment procedure, which must be reviewed and updated on a regular basis. The risk assessment's findings can be used to choose the best course of action, which may include technical measures at first (34). Key tactics for collective prevention include the development and distribution of specialized health-pedagogical training programs. The prevention of skin cancer in outdoor workers is thought to depend heavily on initiatives that include health education, which can help employees better comprehend and perceive the occupational solar UVR risk (13, 34, 39).

Secondary prevention includes screening and early diagnosis as two main components, as stated by the IARC, which shall ultimately result in the early identification of precancerous conditions or malignancies in the beginning stages (35). One of the most important approaches of secondary prevention at work is occupational health surveillance of employees who are exposed to solar UVR and are thus more likely to suffer from subsequent adverse effects. Workers with conditions that may affect their susceptibility to the risk (such as fair skin phototypes I and II, those using hydrochlorothiazide, or those who are under immunosuppression) should be given additional caution. A frequent component of health surveillance involves periodic health examinations of the personnel by licensed occupational health specialists. Additional medical specialists, such as dermatologists, should also be involved in supplemental health management on an individual basis (13, 24, 38).

Tertiary prevention refers to intervention strategies used after adverse effects have already developed. Tertiary preventive measures aim to offer a safe return to work, recovery from the condition, and a good quality of life along with compensation; they further include medical and occupational rehabilitation of employees with UVR-related skin cancers after treatments (15, 38).

## 4. Discussion

A wide range of studies report on high levels of individual solar UVR exposure at work and inadequate adoption of sun-protective activities and routines by outdoor workers (13, 39, 40). The risks of exposure to solar UVR at work are mostly neglected; legal recognition, medical care, and compensation seem, however, to appear as obvious future issues (3). Improved sun protection through legally enforceable laws and regulations is equally pivotal to lowering cancer risks for outdoor workers as general prevention—in this, the role of health professionals is crucial (3). Dermatologists will likely become increasingly important in improving patient care and outcomes in dermato-oncology in the future, especially in light of novel diagnostic and therapeutic approaches for both early and advanced skin cancer, as well as the expanding range of abilities, skills, and knowledge needed to manage this heterogeneous spectrum of diseases (2). Nevertheless, it is crucial that cases are reported to the appropriate authorities to showcase occupational causation. Recently, open access notification forms with a variety of uses for notifying the appropriate authorities of suspected cases were provided (41).

Sadly, to this day, even in the few nations where NMSC is recognized as an occupational disease, affected workers are largely denied the benefits of legal recognition because of the massive underreporting: the responsible physician or rather dermatologist does not notify, as the correlation between the disease and the occupation is not yet routinely made (3). Since skin cancer was added to the list of occupational diseases in 2000, only 36 instances have been reported in Denmark (42). The situation is the same in Italy, where UVR-induced NMSC is listed as one of the country's occupational diseases: Between 2002 and 2017, there were, on average, only 34 instances recorded annually (24); other nations also experienced a major underreporting problem (38). When some types of NMSC (i.e., SCC, multiple AK) were formally recognized in the national decree of occupational diseases in 2015, the situation in Germany changed. More than 7,700 cases of occupational skin cancer were reported in the first 12 months after it was introduced. With 9,931 reports in 2019, skin cancer was the second-most commonly legally recognized disease and the third-most frequently reported occupational disease. It is important to note that a financial incentive has been implemented to encourage physicians to report, which has undoubtedly played a crucial role in the high notification rates. Additionally, patients with recognized occupational skin cancer are given priority medical attention and, in more serious circumstances, significant compensation.

A striking advancement in health and safety laws has been made in Germany as a result of the unexpectedly high UVR exposures among outdoor workers by recent measuring campaigns in this nation (30) and repeatedly in many other countries (14, 18, 25–28). For the first time, as of 12 July 2019, companies are explicitly obliged to carry out a unique solar UVR exposure risk assessment, provide personal protective equipment (plus sunscreens), and give solar UVR-exposed workers a triennial occupational physician consultation (43). The recent German example demonstrates that politicians tend to only act when they receive notifications. In order to combat underreporting and collect better, more accurate disease data worldwide, the 11th edition of the World Health Organization (WHO) International Classification of Diseases (ICD), adopted on May 25, 2019, can be seen as a significant milestone. For the first time, NMSC, including AK, can be categorized as an occupational disease, and BCC and SCC are now distinct entities (44). As a result, ICD 11, implemented in January 2022, will probably show the full epidemiological scope of work-related solar UVR-induced skin cancer and could offer essential new worldwide public health information for preventing cancer among outdoor workers (3). Recent research by Loney et al. (10) has shown that there is currently a dearth of information on skin cancer among employees who are exposed to solar UVR at work in many parts of the world. The WHO and the International Labor Organization (ILO) are actively evaluating the global disease burden of NMSC within the United Nations (UN) Sustainable Development Goals 2030 framework due to the urgency of the growing number of NMSC cases connected to occupational solar UVR exposure. It is included by both UN agencies as one of the top 10 occupational risk factors and health outcomes that are extremely likely to account for a significant burden of disease but have never been taken into consideration in prior global estimating techniques (5, 6).

The urgent need for interventions to improve outdoor workers' sun protection behavior has previously been acknowledged. Whilst designing interventions, the risk perception and attitudes of outdoor workers regarding sun protection measures should be taken into account, as in a recent study it was shown that these variables may have an impact on actual sun protection behavior at work (17). Another recent study discovered that, despite the fact that many occupational groups have shown a tendency to be generally willing to improve their sun protection behavior, the special demands of the outdoor workers are rarely considered (3, 16, 37). Essential new methods for assessing secondary performance attributes [i.e., bio-stability on the skin, eye irritation (burning), absorption time, grip and subjective skin feeling, compatibility with textiles, dust and dirt absorption, and whitening effect] of sunscreens in order to specifically design them for use in the occupational field and thus increase acceptance in professional outdoor work have recently been developed (37). Further, a curriculum in accordance with the “Template for Intervention, Description and Replication” (TIDieR) (45) for multipliers training for prevention of occupational skin cancer in outdoor workers has been presented by Ludewig et al. (46). It seems crucial to further investigate the effectiveness of interventions to prevent occupational skin cancer in outdoor workers, and consequently provide reliable indications for the actual reduction of skin cancer incidence in this professional group (47).

## 5. Conclusion

In conclusion, the complete range of available preventive actions should be utilized for combating occupational skin cancer. This seems pivotal to handle the present and future challenges in a purposeful manner. This will almost definitely only be possible if politicians' support is effectively combined with communal and individual preventive actions in order to spur long-term transformation.

## Data availability statement

The original contributions presented in the study are included in the article/supplementary material, further inquiries can be directed to the corresponding author.

## Author contributions

CS and SJ contributed to conception and design of the paper. CS wrote the first draft of the manuscript. SJ wrote sections of the manuscript. Both authors contributed to manuscript revision, read, and approved the submitted version.

## References

[1] Park YJ Kwon GH Kim JO Kim NK Ryu WS Lee KS. A retrospective study of changes in skin cancer characteristics over 11 years. Arch Craniofac Surg. (2020) 21:87–91. 10.7181/acfs.2020.00024 32380807 PMC7206466

[2] Garbe C Peris K Soura E Forsea AM Hauschild A Arenbergerova M. The evolving field of Dermato-oncology and the role of dermatologists: Position Paper of the EADO, EADV and Task Forces, EDF, IDS, EBDV-UEMS and EORTC Cutaneous Lymphoma Task Force. J Eur Acad Dermatol Venereol. (2020) 34:2183–97. 10.1111/jdv.16849 32840022

[3] John SM Garbe C French LE Takala J Yared W Cardone A. Improved protection of outdoor workers from solar ultraviolet radiation: Position statement. J Eur Acad Dermatol Venereol. (2021) 35:1278–84. 10.1111/jdv.17011 33222341

[4] Trakatelli M Barkitzi K Apap C Majewski S De Vries E. Skin cancer risk in outdoor workers: A European multicenter case-control study. J Eur Acad Dermatol Venereol. (2016) 30(Suppl.3):5–11. 10.1111/jdv.13603 26995016

[5] Paulo MS Adam B Akagwu C Akparibo I Al-Rifai RH Bazrafshan S. WHO/ILO work-related burden of disease and injury: Protocol for systematic reviews of occupational exposure to solar ultraviolet radiation and of the effect of occupational exposure to solar ultraviolet radiation on melanoma and non-melanoma skin cancer. Environ Int. (2019) 126:804–15. 10.1016/j.envint.2018.09.039 30792021

[6] Boniol M Hosseini B Ivanov I Náfrádi B Neira M Olsson A. The Effect of Occupational Exposure to Solar Ultraviolet Radiation on Malignant Skin Melanoma and Nonmelanoma Skin Cancer: A Systematic Review and Meta-analysis From the WHO/ILO Joint Estimates of the Work-related Burden of Disease and Injury. Geneva: World Health Organization (2021).

[7] Peters CE Ge CB Hall AL Davies HW Demers PA. CAREX Canada: An enhanced model for assessing occupational carcinogen exposure. Occup Environ Med. (2015) 72:64–71. 10.1136/oemed-2014-102286 24969047 PMC4283677

[8] McKenzie JF El-Zaemey S Carey RN. Prevalence of exposure to multiple occupational carcinogens among exposed workers in Australia. Occup Environ Med. (2020) 2020:106629. 10.1136/oemed-2020-106629 32948666

[9] Kauppinen T Toikkanen J Pedersen D Young R Ahrens W Boffetta P. Occupational exposure to carcinogens in the European Union. Occup Environ Med. (2000) 57:10–8. 10.1136/oem.57.1.10 10711264 PMC1739859

[10] Loney T Paulo MS Modenese A Gobba F Tenkate T Whiteman DC. Global evidence on occupational sun exposure and keratinocyte cancers: A systematic review. Br J Dermatol. (2021) 184:208–18. 10.1111/bjd.19152 32320481

[11] Armstrong BK Kricker A. The epidemiology of UV induced skin cancer. J Photochem Photobiol B. (2001) 63:8–18. 10.1016/S1011-1344(01)00198-1 11684447

[12] Fitzmaurice C Abate D Abbasi N Abbastabar H Abd-Allah F Abdel-Rahman O. Global, regional, and national cancer incidence, mortality, years of life lost, years lived with disability, and disability-adjusted life-years for 29 cancer groups, 1990 to 2017: A systematic analysis for the global burden of disease study. J Am Med Assoc Oncol. (2019) 5:1749–68. 10.1001/jamaoncol.2019.2996 31560378 PMC6777271

[13] Modenese A Korpinen L Gobba F. Solar radiation exposure and outdoor work: An underestimated occupational risk. Int J Environ Res Public Health. (2018) 15:102063. 10.3390/ijerph15102063 30241306 PMC6209927

[14] Peters CE Pasko E Strahlendorf P Holness DL Tenkate T. Solar ultraviolet radiation exposure among outdoor workers in three Canadian provinces. Ann Work Expo Health. (2019) 63:679–88. 10.1093/annweh/wxz044 31165866

[15] International Commission on Non-Ionizing Radiation Protection. ICNIRP statement—Protection of workers against ultraviolet radiation. Health Phys. (2010) 99:66–87. 10.1097/HP.0b013e3181d85908 20539126

[16] Ludewig M Rocholl M John SM Wilke A. Secondary prevention of UV-induced skin cancer: Development and pilot testing of an educational patient counseling approach for individual sun protection as standard procedure of patient care. Int Arch Occup Environ Health. (2020) 93:765–77. 10.1007/s00420-020-01532-7 32162123 PMC7320965

[17] Rocholl M Ludewig M John SM Bitzer EM Wilke A. Outdoor workers' perceptions of skin cancer risk and attitudes to sun-protective measures: A qualitative study. J Occup Health. (2020) 62:e12083. 10.1002/1348-9585.12083 31478315 PMC6970388

[18] Wittlich M John SM Tiplica GS Sălăvăstru CM Butacu AI Modenese A. Personal solar ultraviolet radiation dosimetry in an occupational setting across Europe. J Eur Acad Dermatol Venereol. (2020) 34:1835–41. 10.1111/jdv.16303 32080895

[19] Schmitt J Haufe E Trautmann F Schulze HJ Elsner P Drexler H. Is ultraviolet exposure acquired at work the most important risk factor for cutaneous squamous cell carcinoma? Results of the population-based case-control study FB-181. Br J Dermatol. (2018) 178:462–72. 10.1111/bjd.15906 28845516

[20] Schmitt J Haufe E Trautmann F Schulze HJ Elsner P Drexler H. Occupational UV-exposure is a major risk factor for basal cell carcinoma: Results of the population-based case-control study FB-181. J Occup Environ Med. (2018) 60:36–43. 10.1097/JOM.0000000000001217 29111985

[21] Bauer A Haufe E Heinrich L Seidler A Schulze HJ Elsner P. Basal cell carcinoma risk and solar UV exposure in occupationally relevant anatomic sites: Do histological subtype, tumor localization and Fitzpatrick phototype play a role? A population-based case-control study. J Occup Med Toxicol. (2020) 15:28. 10.1186/s12995-020-00279-8 32944060 PMC7488106

[22] Bauer A Adam KE Soyer PH Adam KWJ. Prevention of occupational skin cancer. In:SM John JD Johansen T Rustemeyer P Elsner HI Maibach, editors, Kanerva's Occupational Dermatology. Cham: Springer International Publishing. (2020). p. 1685–97.

[23] Watson M Holman DM Maguire-Eisen M. Ultraviolet radiation exposure and its impact on skin cancer risk. Semin Oncol Nurs. (2016) 32:241–54. 10.1016/j.soncn.2016.05.005 27539279 PMC5036351

[24] Gobba F Modenese A John SM. Skin cancer in outdoor workers exposed to solar radiation: A largely underreported occupational disease in Italy. J Eur Acad Dermatol Venereol. (2019) 33:2068–74. 10.1111/jdv.15768 31265157 PMC6899887

[25] Kovačić J Wittlich M John SM Macan J. Personal ultraviolet radiation dosimetry and its relationship with environmental data: A longitudinal pilot study in Croatian construction workers. J Photochem Photobiol B. (2020) 207:111866. 10.1016/j.jphotobiol.2020.111866 32330848

[26] Modenese A Gobba F Paolucci V John SM Sartorelli P Wittlich M. Occupational solar UV exposure in construction workers in Italy: Results of a one-month monitoring with personal dosimeters. In: 2020 IEEE International Conference on Environment and Electrical Engineering and 2020 IEEE Industrial and Commercial Power Systems Europe (EEEIC/I&CPS Europe). Madrid. (2020). 10.1109/EEEIC/ICPSEurope49358.2020.916085227295638

[27] Modenese A Gobba F Paolucci V John SM Sartorelli P Wittlich M. One-month monitoring of exposure to solar UV radiation of a group of construction workers in tuscany. Energies. (2020) 13:6035. 10.3390/en13226035

[28] Moldovan HR Wittlich M John SM Brans R Tiplica GS Salavastru C. Exposure to solar UV radiation in outdoor construction workers using personal dosimetry. Environ Res. (2020) 181:108967. 10.1016/j.envres.2019.108967 31806287

[29] Wittlich M Westerhausen S Kleinespel P Rifer G Stöppelmann W. An approximation of occupational lifetime UVR exposure: Algorithm for retrospective assessment and current measurements. J Eur Acad Dermatol Venereol. (2016) 30(Suppl.3):27–33. 10.1111/jdv.13607 26995020

[30] Wittlich M Westerhausen S Strehl B Schmitz M Stöppelmann W Versteeg H. Exposition von Beschäftigten gegenüber solarer UV-Strahlung - Ergebnisse des Projekts mit Genesis-UV. Berlin: Deutsche Gesetzliche Unfallversicherung e.V. (DGUV) (2020). Available online at: https://publikationen.dguv.de/widgets/pdf/download/article/3993 (accessed November 28, 2022).

[31] Schmitt J Seidler A Diepgen TL Bauer A. Occupational ultraviolet light exposure increases the risk for the development of cutaneous squamous cell carcinoma: A systematic review and meta-analysis. Br J Dermatol. (2011) 164:291–307. 10.1111/j.1365-2133.2010.10118.x 21054335

[32] Bauer A Diepgen TL Schmitt J. Is occupational solar ultraviolet irradiation a relevant risk factor for basal cell carcinoma? A systematic review and meta-analysis of the epidemiological literature. Br J Dermatol. (2011) 165:612–25. 10.1111/j.1365-2133.2011.10425.x 21605109

[33] Breitbart EW Choudhury K Anders MP Volkmer B Greinert R Katalinic A. Benefits and risks of skin cancer screening. Oncol Res Treat. (2014) 37(Suppl.3):38–47. 10.1159/000364887 25195831

[34] Alfonso JH Bauer A Bensefa-Colas L Boman A Bubas M Constandt L. Minimum standards on prevention, diagnosis and treatment of occupational and work-related skin diseases in Europe—Position paper of the COST Action StanDerm (TD 1206). J Eur Acad Dermatol Venereol. (2017) 31(Suppl.4):31–43. 10.1111/jdv.14319 28656728

[35] International Agency for Research on Cancer of the World Health Organization. IARC Handbooks of Cancer Prevention - Preamble for Primary Prevention. Lyon. (2019). Available online at: https://handbooks.iarc.fr/documents-handbooks/hb-preamble-primary-prevention.pdf (accessed November 28, 2022).

[36] Tenkate T Strahlendorf P. Sun Safety at Work: A Management Systems Approach to Occupational Sun Safety. Toronto, ON: Ryerson University (2020).

[37] Rocholl M Weinert P Bielfeldt S Laing S Wilhelm KP Ulrich C. New methods for assessing secondary performance attributes of sunscreens suitable for professional outdoor work. J Occup Med Toxicol. (2021) 16:25. 10.1186/s12995-021-00314-2 34225747 PMC8256554

[38] Ulrich C Salavastru C Agner T Bauer A Brans R Crepy MN. The European Status Quo in legal recognition and patient-care services of occupational skin cancer. J Eur Acad Dermatol Venereol. (2016) 30(Suppl.3):46–51. 10.1111/jdv.13609 26995023

[39] Reinau D Weiss M Meier CR Diepgen TL Surber C. Outdoor workers' sun-related knowledge, attitudes and protective behaviours: A systematic review of cross-sectional and interventional studies. Br J Dermatol. (2013) 168:928–40. 10.1111/bjd.12160 23252833

[40] Schmalwieser AW Casale GR Colosimo A Schmalwieser SS Siani AM. Review on occupational personal solar UV exposure measurements. Atmosphere. (2021) 12:142. 10.3390/atmos1202014227819697

[41] Skudlik C Tiplica GS Salavastru C John SM. Instructions for use of the OSD notification forms. J Eur Acad Dermatol Venereol. (2017) 31(Suppl.4):44–6. 10.1111/jdv.14320 28656729

[42] Carøe TK Ebbehøj NE Wulf HC Agner T. Recognized occupational skin cancer in Denmark—Data from the last 10 years. Acta Derm Venereol. (2013) 93:369–71. 10.2340/00015555-1484 23303569

[43] Federal Ministry of Justice for Consumer Protection. German “Ordinance on Preventive Occupational Health Care (ArbMedVV).” (2019). Available online at: http://www.gesetze-im-internet.de/englisch_arbmedvv/index.html (accessed November 28, 2022).

[44] World Health Organization. WHO Website. Classifications. ICD. (2021). Available online at: https://www.who.int/classifications/icd/en/ (accessed November 28, 2022).

[45] Hoffmann T Glasziou P Boutron I Milne R Perera R Moher D. Better reporting of interventions: Template for intervention description and replication (TIDieR) checklist and guide. Br Med J. (2016) 348:g1687. 10.1136/bmj.g1687 24609605

[46] Ludewig M Rocholl M John SM Wilke A. Prevention of occupational skin cancer in outdoor workers: Development of a curriculum for multipliers training. Präv Gesundheitsf. (2022) 2022:6. 10.1007/s11553-022-00940-6

[47] Modenese A Loney T Rocholl M Symanzik C Gobba F John SM. Protocol for a systematic review on the effectiveness of interventions to reduce exposure to occupational solar ultraviolet radiation (UVR) among outdoor workers. Front Public Health. (2021) 9:756566. 10.3389/fpubh.2021.756566 34858932 PMC8632259

